# On‐Surface Assembly of Hydrogen‐ and Halogen‐Bonded Supramolecular Graphyne‐Like Networks

**DOI:** 10.1002/anie.201916708

**Published:** 2020-04-01

**Authors:** Zechao Yang, Lukas Fromm, Tim Sander, Julian Gebhardt, Tobias A. Schaub, Andreas Görling, Milan Kivala, Sabine Maier

**Affiliations:** ^1^ Department of Physics Friedrich-Alexander-Universität Erlangen-Nürnberg Erwin-Rommel-Str. 1 91058 Erlangen Germany; ^2^ Chair of Theoretical Chemistry Department of Chemistry and Pharmacy Friedrich-Alexander-Universität Erlangen-Nürnberg Egerlandstr. 3 91058 Erlangen Germany; ^3^ Max Planck Institute for the Structure and Dynamics of Matter Department 22761 Hamburg Germany; ^4^ Organisch-Chemisches Institut Ruprecht-Karls-Universität Heidelberg Im Neuenheimer Feld 270 Heidelberg Germany; ^5^ Centre for Advanced Materials Ruprecht-Karls-Universität Heidelberg Im Neuenheimer Feld 225 Heidelberg Germany

**Keywords:** graphyne, halogen bonds, hydrogen bonds, nanostructures, self-assembly, surface chemistry

## Abstract

Demonstrated here is a supramolecular approach to fabricate highly ordered monolayered hydrogen‐ and halogen‐bonded graphyne‐like two‐dimensional (2D) materials from triethynyltriazine derivatives on Au(111) and Ag(111). The 2D networks are stabilized by N⋅⋅⋅H−C(sp) bonds and N⋅⋅⋅Br−C(sp) bonds to the triazine core. The structural properties and the binding energies of the supramolecular graphynes have been investigated by scanning tunneling microscopy in combination with density‐functional theory calculations. It is revealed that the N⋅⋅⋅Br−C(sp) bonds lead to significantly stronger bonded networks compared to the hydrogen‐bonded networks. A systematic analysis of the binding energies of triethynyltriazine and triethynylbenzene derivatives further demonstrates that the X_3_‐synthon, which is commonly observed for bromobenzene derivatives, is weaker than the X_6_‐synthon for our bromotriethynyl derivatives.

## Graphical abstract

**The on‐surface assembly** of hydrogen‐ and halogen‐bonded supramolecular graphyne‐like networks from functionalized triethynyltriazine derivatives on metal surfaces, Au(111) and Ag(111), is described. The networks are stabilized by N⋅⋅⋅H−C(sp) bonds and N⋅⋅⋅Br−C(sp) bonds to the triazine core.

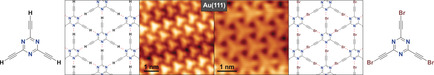

## Introduction

Graphyne is an sp–sp^2^‐hybridized carbon allotrope that is composed of periodic acetylene (‐C≡C‐) linkages connecting aromatic benzene branching units.^1^ Because of the well‐ordered porous structure, where the triple bonds provide chemisorption sites for metal adatoms and small molecules,^2^ the presence of a natural band gap,^3^ and the high degree of π‐conjugation,^4^ graphyne is expected to be an even more exciting material than graphene for applications in nanoelectronics, next‐generation batteries, hydrogen‐storage systems, and sensor devices.^5^ However, strategies for the reliable synthesis of single‐layer crystalline graphyne remain elusive.^6^ The bottom‐up synthesis on surfaces provides a versatile approach for the growth of two‐dimensional (2D) materials to fabricate novel carbon‐based nanostructures that cannot be obtained by conventional solution chemistry. One of the key issues scientists presently are facing is the control of the alkyne coupling reactions on metal surfaces.^7^ The on‐surface synthesis of graphyne‐based structures that are directive in fabricating extended graphyne networks therefore attracted great interest recently.^8^

**Figure 1:**
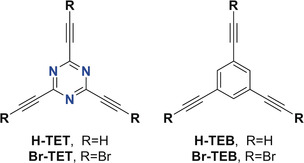
Chemical structures of the investigated 2,4,6‐triethynyl‐1,3,5‐triazine (TET) and 1,3,5‐triethynylbenzene (TEB) derivatives.

Herein, we report the on‐surface assembly of 2D networks from 2,4,6‐triethynyl‐1,3,5‐triazine (TET) and 1,3,5‐triethynylbenzene (TEB) derivatives (Figure 1) on Ag(111) and Au(111). The TET‐based networks may be regarded as stable supramolecular analogues of γ‐graphyne. We employ scanning tunneling microscopy (STM) in combination with density‐functional theory (DFT) to demonstrate that the triazine core enables stable hydrogen (H‐bonds) and halogen bonds (X‐bonds) between the N_triazine_ and the H‐ and Br‐terminated acetylenes, respectively. Thereby, the supramolecular graphyne networks based on N⋅⋅⋅Br−C(sp) bonds to the triazine core, which are known from supramolecular chemistry^9^ but hitherto unstudied in self‐assemblies on surfaces in ultra‐high vacuum, have a significantly stronger binding energy than the previously reported N⋅⋅⋅H−C(sp)‐bonded networks.^10^ While for the triethynyltriazine derivatives, dense hexagonal close‐packed (hcp) structures are thermodynamically favored, hexameric structures of six interacting Br‐terminal groups (X_6_‐synthon)^11^ dominate when substituting the triazine by a benzene ring. It is important to note, however, that the noncovalently ordered hcp structures do not directly convert into covalently linked N‐containing graphdiyne upon reaction, as demonstrated at the gas/liquid and liquid/liquid interface for several layered 2D materials.^12^ In contrast, on the surface, the reorientation of the Br‐TET upon debromination results in the honeycomb Ag‐bis(acetylide) networks reported previously.^13^

## Results and Discussion

### Hydrogen‐Bonded Graphyne‐Like 2D Networks

**Figure 2:**
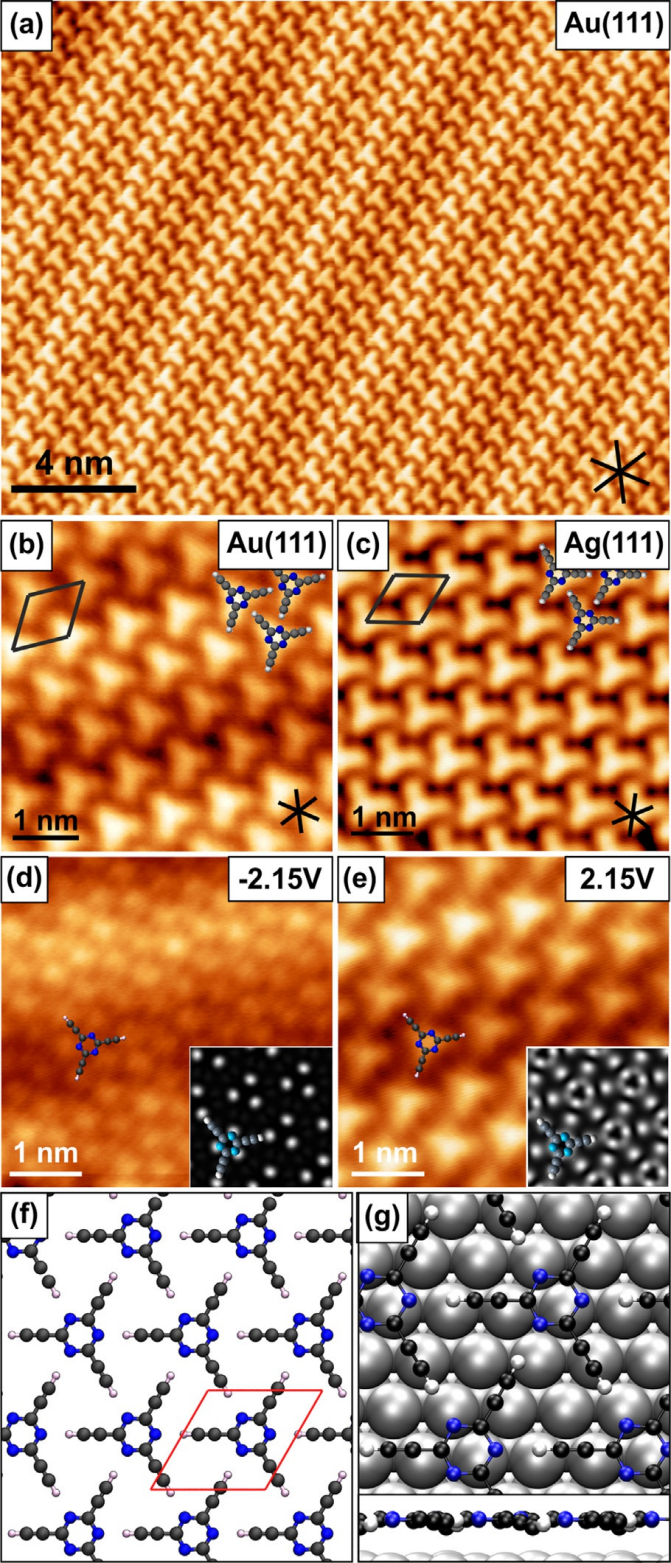
STM images of self‐assembled H‐TET on a,b) Au(111) and c) Ag(111) upon deposition at RT. d,e) Filled‐ and unfilled‐state STM images of H‐TET on Au(111). The corresponding calculated STM images from optimized gas‐phase structures for −0.35 V and 4.85 V are shown as an inset. The shift towards negative energies in the experiment compared to DFT in gas phase originates from a charge transfer from the surface to the H‐TET‐networks, which is consistent with the one previously reported for triethynyltriazine derivatives on Ag(111).^13^ f,g) DFT‐optimized H‐TET hcp structure (g) on Ag(111) and (f) free‐standing. STM parameters: a) *U*=0.3 V, *I*=30 pA; b,c) *U*=0.2 V, *I*=40 pA. The black lines in the right lower corner in the STM images indicate the close‐packed lattice directions of the metal substrate. Color code: carbon, dark gray; nitrogen, blue; silver, light gray; hydrogen, white.

Upon deposition of submonolayer coverage of H‐TET on Au(111) and Ag(111) at 300 K, extended 2D self‐assemblies were observed by STM (Figure 2 a). The high‐resolution STM images (Figure 2 b,c) reveal that the triangular‐shaped molecules assemble in an hcp structure with each ethynyl group pointing to the triazine core of the neighboring molecule. Based on the rhombic unit cell with dimensions of *a*=*b=*0.87±0.05 nm and *θ*=120±3° as determined from STM analysis, the H‐bond N_triazine_⋅⋅⋅H−C(sp) is estimated to be around 2.3 Å in length, which is consistent with the molecular crystal structure (2.31 and 2.34 Å).^10^ We note that the apparent shape of H‐TET strongly depends on the applied bias voltage because of a convolution of the molecule's electronic properties and the topography at higher bias voltages in STM (Figure 2 d,e). The filled‐state STM images reveal the triazine core (Figure 2 d), while in the gap and for unfilled states the images (Figure 2 e) depict the full shape of the H‐TET molecule. The observed image contrasts are in excellent agreement with calculated STM images in the gas phase, and on the surface, respectively (see also Figure S1 in the Supporting Information).

Besides, a nonperiodic second phase was also found on the Au(111) and Ag(111) surface, which we identified as reacted molecules and impurities (see Figure S2). In conclusion, the self‐assembled H‐TET networks are mainly governed by H‐bond interactions. They can be considered as a hydrogen‐bonded supramolecular analogue to γ‐graphyne similar to the previously reported layered hexagonal network structures formed through the supramolecular self‐assembly of H‐TET bulk structures in the solid state.^10^

There are several orientations of the almost planar H‐TET with respect to the surface lattice found, in which an adsorption of the acetylenic side groups along the high‐symmetry axis of the substrate is the most frequent. This finding points to a weak influence of the underlying substrate on the adsorption behavior of H‐TET, which is also confirmed by DFT. DFT calculations of H‐TET adsorbed on Ag(111) reveal that the molecules are adsorbed mostly flat with an adsorption distance of 3.00 Å (Figure 2 g), which is closer than on Au(111).^14^ However, the acetylenic units are slightly bent towards the nearest Ag surface atom, leading to a maximal corrugation of 0.05 Å. This attractive interaction results in an adsorption distance that is about 0.16 Å smaller than for an isolated triazine molecule adsorbed on Ag. It also increases the adsorption energy by about 0.60 eV. Despite this energy increase, the adsorption site of the H‐TET polymer with respect to the surface lattice is rather insensitive. The different structures we tested yielded similar adsorption distances within 0.03 Å and adsorption energies within 0.03 eV (see Figure S3), in line with the rather large adsorption distance that is known for physisorption of graphene^15^ and related covalently linked networks.^16^ All this data confirms the weak interaction of H‐TET with the noble metal surfaces and explains why the structural data for the computed structures in vacuum in Table SI1 is in excellent agreement with the experimental results. Moreover, this corroborates the small variance of the experimental H‐TET structures on Au(111) and Ag(111) compared to the molecular crystal^10^ within 0.2 Å.

### Halogen‐Bonded Graphyne‐Like 2D Networks

**Figure 3:**
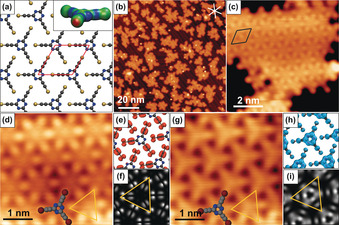
Br‐TET on Au(111) upon deposition at 90 K. a) Structural model from DFT of halogen‐bonded graphyne‐like networks. The corresponding electrostatic potential distributions of Br‐TET in the inset shows the positive potential in red and the negative potential in blue at isodensity surfaces. b–d,g) STM images of halogen‐bonded graphyne‐like networks. The high‐resolution images at (d) *U*=−0.2 V and (g) *U*=1.8 V demonstrate the bias‐dependent STM topography with the corresponding (e,h) partial density of states and f,i) calculated STM images at −0.6 V and +2.5 V that provide a good match to the experiment. The shift towards negative energies in the experiment compared to DFT in gas phase originates from a charge transfer from the surface to the Br‐TET‐networks.^13^ STM parameters: b) *U*=−1.0 V, *I*=50 pA; c) *U*=−1.0 V, *I*=30 pA; d) *U*=−0.2 V, *I*=100 pA; g) *U*=1.8 V, *I*=100 pA. Color code: carbon, dark gray; nitrogen, blue; silver, light gray; bromine, brown.

As the next step, we change from H‐ to X‐bonding interactions by studying Br‐TET, which can be derived from H‐TET by exchanging terminal acetylenic H with Br. Thereby, the self‐assembly of Br‐TET is driven by competing interactions, which results in several possible patterns: N_triazine_⋅⋅⋅Br−C(sp) bonding leads to hcp networks, while Br⋅⋅⋅Br halogen bonds would facilitate networks with X_3_‐ or X_6_‐synthon geometries. N_triazine_⋅⋅⋅Br−C(sp) halogen bonding can occur because of electrostatic interactions between the negative electrostatic potential at the N_triazine_ sites and the positive σ‐hole^17^ at Br (see inset Figure 3 a).

The Br−C(sp) bonds cleave on Ag(111) and Au(111) surfaces already below room temperature.^13^ Therefore, Br‐TET was deposited on Au(111) at 90 K, which forms small self‐assembled islands at submonolayer coverage (Figure 3 b,c). Three bright protrusions per molecule with a separation of *a*=1.00±0.05 nm are visible at low bias voltages (see the yellow triangle in Figure 3 d), which correspond to the Br groups and confirm that Br‐TET adsorbs intact on Au(111) at these conditions. The hcp structure of the Br‐TET self‐assembly is qualitatively the same as for H‐TET but formed by N_triazine_⋅⋅⋅Br−C(sp) halogen‐bonded instead of hydrogen‐bonded molecules. The unit cell with dimensions of *a*=*b*=1.00±0.05 nm and *θ*=120±3° is slightly larger than for H‐TET because of the larger van der Waals radius of the Br moieties compared to H. The Br⋅⋅⋅N_triazine_ distance measures about 3 Å, which is typical for a halogen‐bonded N‐heterocycle with Br.^18^ Up to now, STM studies on the formation of 2D molecular networks based on halogen bonds between N‐heterocycles and halogens are elusive because of competing halogen–halogen interactions.^19^ We note that the sterically unbiased ethynyl groups allow us to engineer such assemblies, whereas in 4‐bromophenyl‐substituted N‐heterocycle compounds, halogen–halogen interactions dominate because of steric demands.^20^

The STM contrast of Br‐TET on Au(111) changes significantly around +2 V, indicating the presence of an electronic state at this energy (Figure 3 d,g). Local density of states (LDOS) maps (Figure 3 e,h) and calculated STM images (Figure 3 f,i) reveal that at −0.2 V (Figure 3 d) the Br‐terminal and ethynyl groups dominate the STM contrast, with only small density of states at the triazine core. In contrast, at +1.8 V the LDOS is centered on the triazine core, which gives the impression of an inverted orientation of the molecule in the STM image as highlighted by the overlaid model in Figure 3 g. The orientation is further supported by the edge termination of Br‐TET islands, as seen in Figure S4. The stronger binding energy of −0.94 eV and a band gap of 2.7 eV for the Br‐TET network in comparison to the −0.71 eV binding energy and 3.4 eV band gap for H‐TET, demonstrate that the X‐bonded network is more strongly bound than the H‐bonded supramolecular network (see discussion below).

### Tris(bromoethynyl)‐benzene Networks with an X_6_‐Synthon

**Figure 4:**
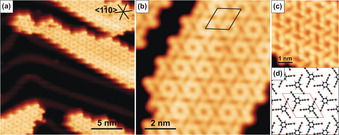
Br‐TEB on Au(111) after deposition at 90 K. a–c) Overview and zoomed‐in STM images of self‐assembled Br‐TEB. d) DFT‐optimized model of the X_6_‐synthon structure. STM parameters: a) *U*=−1.0 V, *I*=40 pA; b) *U*=0.1 V, *I*=300 pA; c) *U*=0.05 V, *I*=20 pA.

We now investigate the change of the self‐assembly replacing Br‐TET with Br‐TEB. This substitution disables nitrogen–halogen bonds between the terminal bromine and the aromatic monomer center by substituting N with C−H, and changes the steric situation close to the benzene core. The Br⋅⋅⋅Br interaction can facilitate Br‐TEB networks with cyclic X_3_‐ or X_6_‐synthon geometries. STM overview images (Figure 4 a,b) show the formation of elongated Br‐TEB islands upon deposition on Au(111) at 90 K, which consist of regularly ordered ringlike features (Figure 4 b). Molecular vacancies (see Figure S5) allow the unambiguous identification of single molecules within the self‐assembly and show that the rings are constructed by six Br moieties of six individual molecules in an X_6_‐synthon geometry (Figure 4 d). Each bromine seems to interact through Br⋅⋅⋅Br halogen bonds with two neighboring molecules. This geometry is an unexpected one compared to a possible variant that would be connected by the more commonly found X_3_‐synthon. The unit cell of the X_6_‐synthon self‐assembly has dimensions of *a*=*b*=1.50±0.05 nm, *θ*=120±3° and is oriented 20±3° with respect to the high‐symmetry directions of the Au(111) surface. At low bias voltages, the molecules can be identified clearly (Figure 4 c). The intramolecular Br–Br distance of *d=*0.98±0.05 nm (compared to 1.01 nm in DFT) confirms that the molecules are intact, which is consistent with the unperturbed herringbone reconstruction of the Au substrate.^21^ This finding suggests that the removal of the N substituent does not destabilize the Br−C(sp) bond significantly. Moreover, the hexameric X_6_‐synthon leads to an organizational chirality with observed homochiral left‐ and right‐handed domains (see Figure S5).

### Intermolecular Interactions in Triethynyl Triazine and Triethynyl Benzene Networks

**Figure 5:**
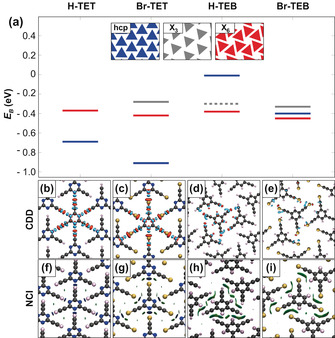
a) Binding energies for H‐TET, Br‐TET, H‐TEB, and Br‐TEB comparing the hcp (blue), X_6_‐synthon (red), X_3_‐synthon (gray), and dense X_3_‐synthon (dashed gray line). b–e) Charge density differences (CDD) and f–i) non‐covalent interaction (NCI) plots of the hcp H‐TET, hcp Br‐TET, X_6_‐synthon H‐TEB and X_6_‐synthon Br‐TEB, respectively. Charge accumulation and depletion are displayed by cyan and orange in the CDD plots. In NCI plots, attractive, repulsive, and weak non‐covalent interactions are represented by a blue, red, and green color.

To get further insight into the different interactions and to discuss the two modifications (nitrogen substitution and terminal group variation), we now compare DFT calculations of the 2D networks of H‐TET, Br‐TET, Br‐TEB, and H‐TEB. We compare up to four structural models: the compact hcp assemblies, the hexameric X_6_‐synthon, and the more commonly found X_3_‐synthon with two different packing densities. Figure 5 shows the relative thermodynamic preference for each case. We find that the hcp structure is the favorable structure for the TET‐based monomers, with the Br‐terminated modification (Br‐TET) being more stable than H‐TET (0.22 eV per monomer). This trend is also observed for the X_6_‐synthon structure, which is less stable for both terminations. A possible X_3_‐synthon is further destabilized for Br‐TET, suggesting that the interactions in the X_6_‐synthon structure go beyond halogen–halogen bonds. For H‐TET, such an X_3_‐structure is just hypothetical since the strong N–H hydrogen bond drives the system to the hcp structure without a local minimum. Substitution of C−H for N in the TEB based networks disables the stabilizing hydrogen or halogen bonds to the N center and instead the space is occupied by an additional hydrogen atom to which only weak intermolecular interactions are possible. This steric interference destabilizes the hcp structure and the X_6_‐synthon becomes the most stable structure, explaining the experimentally observed changes. The binding strength in the X_6_‐synthon is comparable for TET‐ and TEB‐based molecules, but for the TEB molecules, the hcp structure is less stable. On top of this, breaking the symmetry in the hcp structure leads to another structure, a denser X_3_‐structure, in which the H/Br‐terminated acetylenes interact with each other. This structure is only stable for TEB‐based molecules.

Comparing the center‐to‐center distance *d*_c‐c_ for the TET‐based hcp structures shows that the H‐terminated structure is by 1.6 Å smaller and therefore, the structure is denser than the Br‐terminated one, in line with a shorter N–H to N–Br distance. This difference does not indicate a weakening of the halogen compared to the hydrogen bond but is solely a consequence of the larger size of the Br atom. Because of the large size of bromine, there are not only interactions directly to the nitrogen of the core but also interactions to the sp‐hybridized triple bonded carbon atoms, which can also be seen in the NCI plot (Figure 5 f,g).

To examine the intermolecular interactions, charge density difference (CDD) and noncovalent interaction (NCI) analysis are performed (Figures 5 b–i; see Figure S6). The stable hcp structures (Figure 5 b,c) clearly show the hydrogen and halogen bonds of the TET networks, with charge redistribution from the terminal groups (H or Br, respectively) to the nitrogen atoms. The TEB networks do not allow this interaction, because of the change in polarity of the CH group and the, therefore, decreased electrostatic interactions. The strong attractive interactions can also be visualized by the NCI plot in Figure 5 f,g.

For the X_6_‐synthon structures (Figure 5 d,e), we do not observe significant interactions between the terminal H or Br atoms of neighboring molecules. Instead, the bonding is dominated by mutual interactions of each terminal group with the neighboring molecule's carbon triple bonds in the arm. In the resulting structure of Br‐TEB, we observe attractive interactions between Br and the neighboring carbon triple bond, which accumulates charge from the halogen. This observation agrees with the presence of a σ‐hole,^17^ which is observed for halogen bonding. In contrast, the X_6_‐structures (see Figure S6) of the TET molecules show bonds of the terminal atoms to the nitrogen center of the core. These bonds are weaker compared to the hcp structures since longer intermolecular distances indicate a weaker binding for the same molecules. This observation is also in line with the decreased intermolecular binding energy.

Last, we investigate the intermolecular interactions in the X_3_‐synthon structures to gain a more complete understanding. For H‐terminated acetylenes, the X_3_‐synthon is not even metastable because no attractive H_3_‐synthon bonding motif exists. In contrast, the Br_3_‐synthon exhibits the expected charge density redistribution between halogen pairs. Because of the increased intermolecular distance and the reduction of bonding interactions per monomer by a factor of two compared to hcp and X_6_‐synthon structures, the X_3_‐synthon is in this case energetically unfavorable. In the case of Br termination, interestingly, the X_6_‐synthon structures and halogen–acetylene bonded networks (hcp) have similar binding energies, despite the significant different bonding motif observed in the CDDs (see Figure S6). Therefore, it can be well understood that the denser X_6_‐synthon (*d*_c‐c_: 8.7 Å vs. 10.9 Å), which has more intermolecular bonds per monomer, is observed to be thermodynamically favored.

## Conclusion

In conclusion, we report the formation of supramolecular hydrogen‐ and halogen‐bonded graphyne‐like networks from triethynyltriazine derivatives on Ag(111) and Au(111). The graphyne‐like networks are based on N⋅⋅⋅Br−C(sp) bonds, which have been elusive in UHV surface studies so far, and N⋅⋅⋅H−C(sp) bonds, respectively. Interestingly, the halogen‐bonded network is significantly more strongly bonded than the N⋅⋅⋅H−C(sp) networks observed previously in the solid state.^10^ We demonstrate that not only the termination of the ethynyl group but also the composition of the molecular core affect the intermolecular interaction and, thus, the structure of the noncovalently linked networks. For the H‐ and Br‐functionalized triethynyl derivatives, we observe a change from hcp to X_6_‐synthon networks, when switching from a triazine core to a benzene core. The halogen–halogen bound X_3_‐synthon, which is commonly observed for bromobenzene derivatives,^22^ is found to be less stable for both functionalized triethynyltriazines and triethynylbenzenes. Our systematic study on the energetics and intermolecular bonding properties of each particular building block on metal surfaces does not only demonstrate the fabrication of supramolecular hydrogen‐ and halogen‐bonded graphyne‐like networks and reveal a novel strong halogen bonding motif on surfaces, but more importantly, it is expected to have a substantial impact on the molecular design for the bottom‐up construction of future acetylenic 2D materials.

## Experimental Section

**STM experiments**. The experiments were performed in a two‐chamber ultra‐high vacuum system, which operates at a base pressure below 1*×*10^*−*10^ mbar. All STM measurements were conducted with a combined low‐temperature scanning tunneling/atomic force microscope from Scienta‐Omicron GmbH in constant‐current mode at 4.7 K using a platinum‐iridium tip. The indicated bias voltages refer to the sample, although the tip was biased and the sample electronically grounded during the STM measurements. The STM images were processed and analyzed using the WSxM software.^23^

The Ag(111) and Au(111) surfaces were cleaned in situ by repeated cycles of Ar^+^ ion sputtering and annealing at 750 K. Br‐TEB, Br‐TET, and H‐TET molecules were evaporated onto the metal substrates from a commercial Knudsen cell (Kentax GmbH) with the quartz crucibles held at 305 K, 390 K, and 320 K, respectively. The molecules were thoroughly degassed several hours before deposition on the surface. The deposition rate of Br‐TEB, Br‐TET, and H‐TET could not be monitored using a quartz crystal microbalance. Instead, an evaporation pressure of around 2×10^*−*10^ mbar was used for calibration of the molecular coverage, which corresponded to a rate of around 0.05 ML min^−1^ according to STM overview images.

**Chemical Synthesis**. The synthesis of 2,4,6‐tris(bromoethynyl)‐1,3,5‐triazine (Br‐TET) is described in Yang et al.^13^ 1,3,5‐Tris(bromoethynyl)benzene (Br‐TEB) was synthesized from known 1,3,5‐triethynylbenzene^24^ (H‐TEB) and 2,4,6‐tris[(trimethylsilyl)ethynyl]‐1,3,5‐triazine^10^ through bromination with N‐bromosuccinimide.

## Computational Methods

DFT calculations were performed with the VASP software package V5.4.1.^25^ using the PBE functional^26^ and a projector augmented plane wave basis set (PAW)^27^ with an energy cutoff of 450 eV (550 eV for cell relaxations). To account for van der Waals interactions the DFT‐D3 correction Scheme by Grimme^28^ was applied with the Becke‐Johnson damping.^29^ Energies and geometry optimizations were converged to 10^*−*7^ eV and forces acting on ions below 0.005 eV Å^−1^, respectively. Free‐standing systems in vacuum were computed with 15 Å separating periodic mirror images into the *z*‐direction; thereby, every cell contains one molecule for the hcp‐, X_3_‐synthon‐ and two molecules for the X_6_‐synthon‐structures which leads to cells of about 8.5 to 16.5 Å in each direction. For all structures, a 5*×*5*×*1 Monkhorst‐Pack grid was used. Calculations of the H‐TET self‐assembly on silver were carried out using a (3*×*3) replica of an optimized Ag(111) (1*×*1) slab containing six layers, fixing the bottom three to their bulk positions. A $\left(2/\;3\sqrt{3}\times 2/\;3\sqrt{3}\right)$ R30° overlayer of H‐TET fits almost perfectly on this silver slab, with a small mismatch of only 0.07 Å (0.85 %). Isolated H‐TET and triazine molecules were considered in a (6*×*6) silver cell. Due to the metallic character of these systems, a first‐order Methfessel‐Paxton level broadening^30^ with *σ*=0.2 eV was used. To account for the finite size of the slab model, a dipole correction^31^ was employed in the *z*‐direction. Intermolecular binding energies per monomer are defined as *E*_ads_=(*E*(*n* M))−*n* *E*(M))/*n*, i.e., subtracting the energy of the isolated monomer *E*(M) from the two‐dimensional network *E*(*n* M) containing *n* monomers. In cases of metal‐supported systems, the isolated metal slab was subtracted as well. Charge density differences were computed alike by subtracting respective charge densities *ρ*_CDD_=*ρ*(*n* M)−(*n*−1)*ρ*(M). The non‐covalent interaction plots^32^ were produced with the program *critic2*. Iso‐densities of the reduced‐density gradient |∇*ρ*|/*ρ* ^4/3^, color‐coded alongside the electron density *ρ* were evaluated between one molecule of the self‐assembly and the rest. In addition, STM images were simulated using the constant height mode, with the tip distance in brackets, in the *p4vasp* program for partial electron densities calculated as described in the *VASP*‐manual (STM of graphene) with the respecting energy values versus the Fermi level.

## Conflict of interest

The authors declare no conflict of interest.

## Supporting information

As a service to our authors and readers, this journal provides supporting information supplied by the authors. Such materials are peer reviewed and may be re‐organized for online delivery, but are not copy‐edited or typeset. Technical support issues arising from supporting information (other than missing files) should be addressed to the authors.

Supplementary
