# Supplementary material for: On‐Surface Assembly of Hydrogen‐ and Halogen‐Bonded Supramolecular Graphyne‐Like Networks
Source: Angew Chem Int Ed Engl. 2020 Apr 1;59(24):9549–55. doi: 10.1002/anie.201916708 (PMC7318139; doi:10.1002/anie.201916708)
Supplement: Supplementary file 1 — Supplementary [file ANIE-59-9549-s001.pdf]

1 Additional STM experiments and DFT calculations

1.1 H-TET

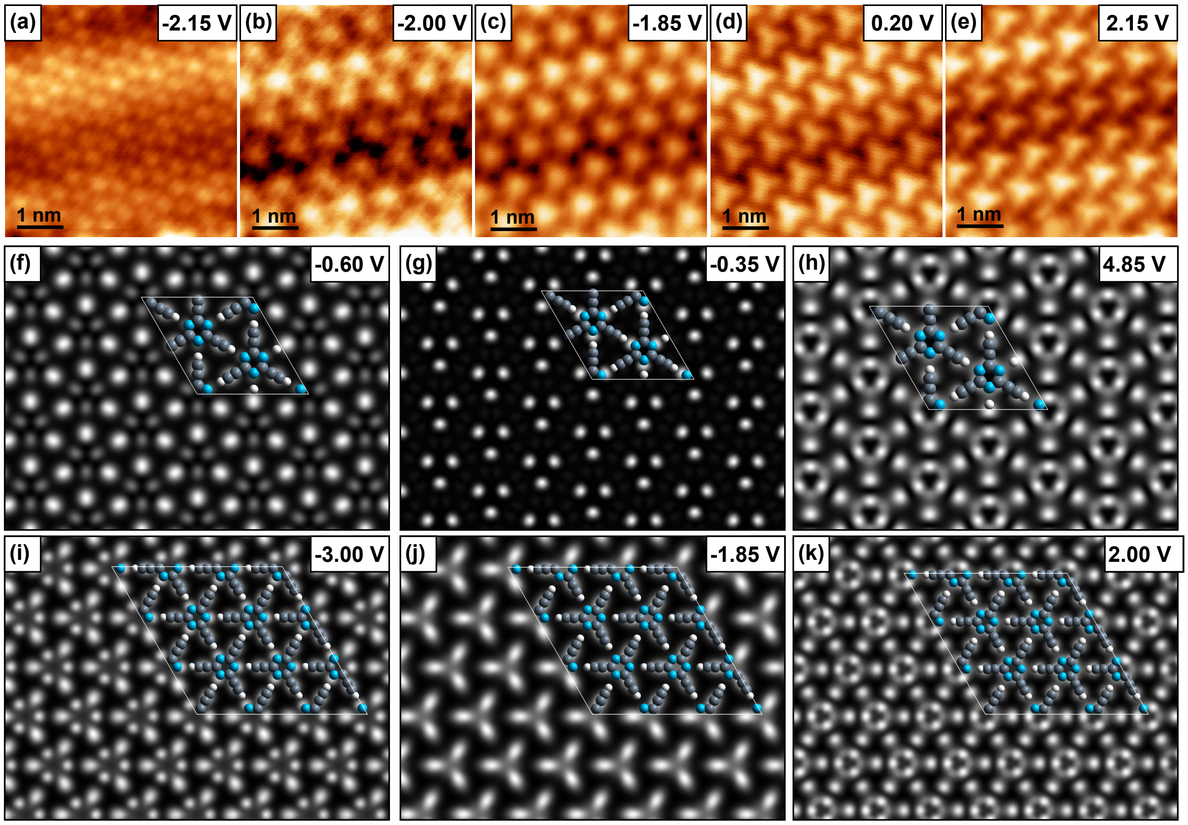

Figure S1: (a-e) STM images of H-TET adsorbed on Au(111) at room temperature measured at different bias voltages. (f-h) Corresponding calculated STM images on the optimized structural model in the gas phase. The calculated filled state images show strongly the (f) ethynyl groups (-0.6 V) and (g) triazine core (-0.35 V). In the experimental STM images, the triazine cores start to appear pronounced below -1.85 V and are clearly seen at images recorded at -2.15 V. The calculated (4.85 V) and measured unfilled state images depict the entire carbon scaffold. The shift towards negative energies of the experimental images compared to calculated gas phase structures originates from a charge transfer from the surface to the H-TET-networks that is consistent with previously reported triethynyltriazine derivatives on Ag(111).[1] (i-k) Calculated STM images for optimized structures on the Ag(111) surface. (i) The filled state image at -3 V reveals a pronounced contrast at the ethynyl groups and triazine core, similar to (f) and (g) in gas phase. The calculated (j) *in-the-gap* image at -1.85 V and (k) unfilled state image at 2.0 V show the entire molecule, which is consistent with the STM images shown in (c-e). In summary, the calculated STM images in gas phase and on the surface explain qualitatively the experimental STM images of the hydrogen bonded graphyne-like H-TET networks well. STM parameter: (a-e) $I = 100$ pA.

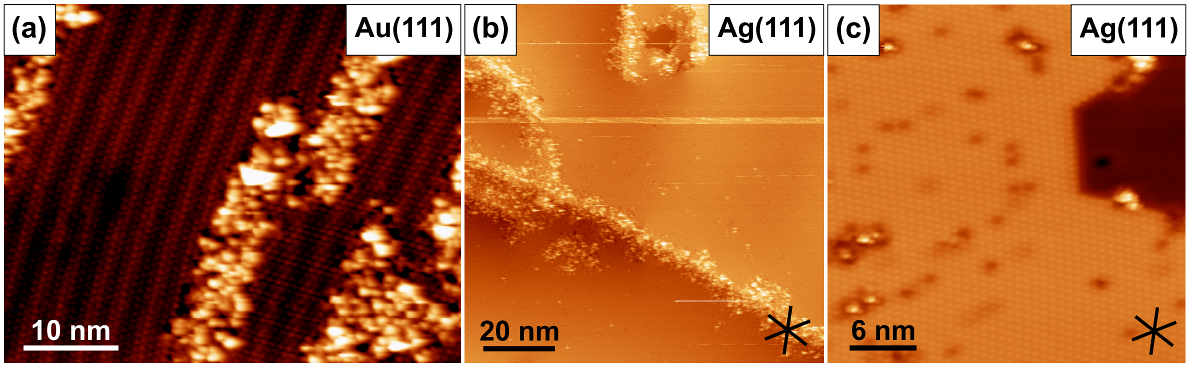

Figure S2: (a) Overview STM image of H-TET self-assemblies on Au(111) upon adsorption at room temperature showing well-ordered H-bonded networks. Besides, a non-periodic second phase was also found, which has an apparent height of around 3 Å. This suggests that the molecules do not adopt an upright adsorption geometry. Instead, the molecules likely start to react locally, forming an unordered phase. The herringbone reconstruction of the Au(111) substrate underneath the ordered self-assembly is unperturbed by the molecules. (b-c) Overview STM images of H-TET on Ag(111) showing well-ordered H-bonded networks. On Ag(111), a non-periodic second phase similar than on Au was observed at some places, see (b), which could be attributed to impurities or reacted molecules. STM parameters: (a) $U = 0.3$ V, $I = 30$ pA; (b) $U = 0.2$ V, $I = 40$ pA, (c) $U = 0.3$ V, $I = 100$ pA. The lines in the right lower corner depict the high-symmetry axes of the Ag(111) surface.

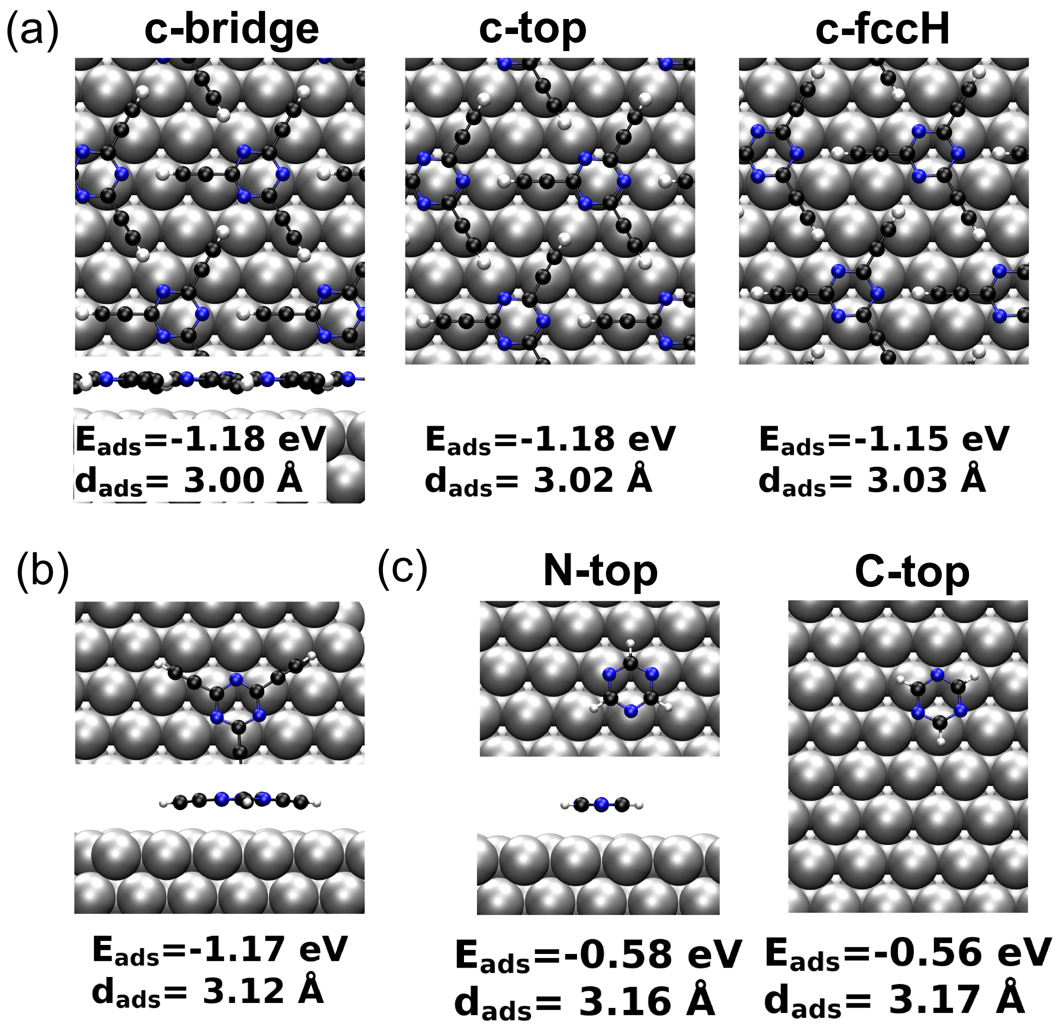

Figure S3: Geometries, adsorption energies, and averaged adsorption distances calculated by DFT for (a) self-assembled H-TET, (b) H-TET monomer, and (c) triazine adsorbed on Ag(111). Color code: carbon, dark gray; nitrogen, blue; silver, light gray; hydrogen, white.

1.2 Br-TET

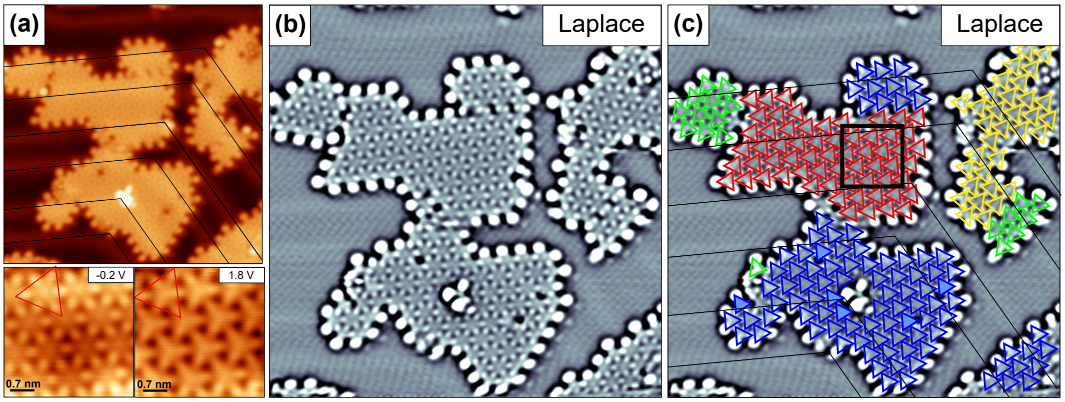

Figure S4: Br-TET on Au(111) after deposition at 90 K. (a) Overview STM image with zoomed-in images recorded at different bias voltages. The red triangles highlight the orientation of the Br-TET and the black lines the unperturbed herringbone reconstruction of the Au(111) surface. (b-c) Laplace-filtered image of (a). In (c) the colored triangles indicate domains with different orientations of Br-TET. The black frame marks the position of the high-resolution STM images shown in (a) and Fig. 3d,g of the manuscript.

1.3 Br-TEB

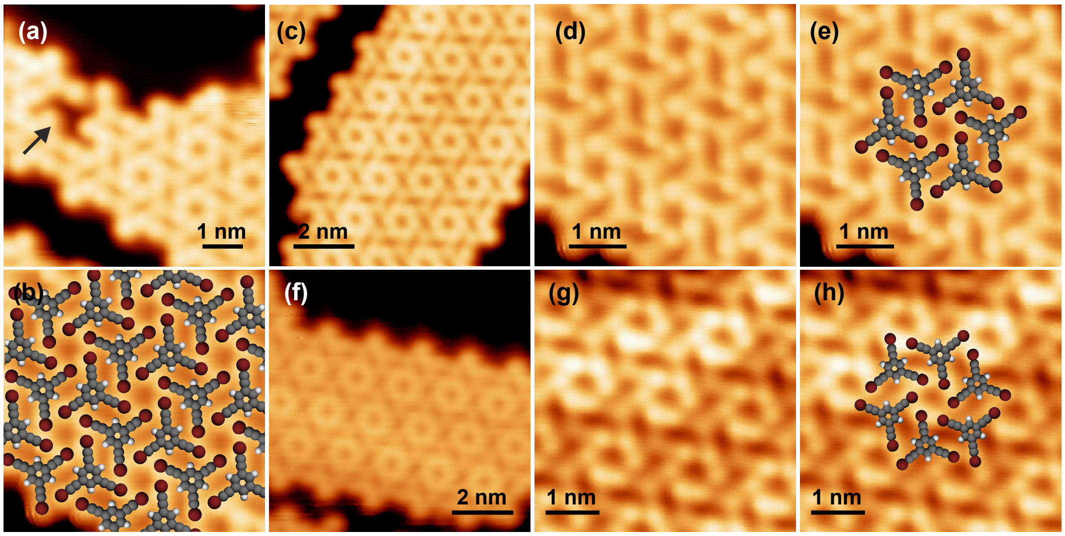

Figure S5: Br-TEB on Au(111) after deposition at 90 K. (a,b) The shape of a single molecule can be identified (a) by a single-molecule vacancy, as marked by the black arrow, and (b) by the edge structure of an island. (c-h) The self-assembly shows organizational chirality, where as (c-e) shows a homochiral left-handed domain and (f-h) a right-handed domain. STM parameters: (a) $U = 1.0$ V, $I = 30$ pA; (b,e) $U = 0.05$ V, $I = 20$ pA; (c) $U = 0.1$ V, $I = 300$ pA; (d) $U = 0.05$ V, $I = 20$ pA; (f) $U = 1.0$ V, $I = 120$ pA; (g,h) $U = 0.05$ V, $I = 700$ pA.

2 CDD analysis, NCI plots, and structural data

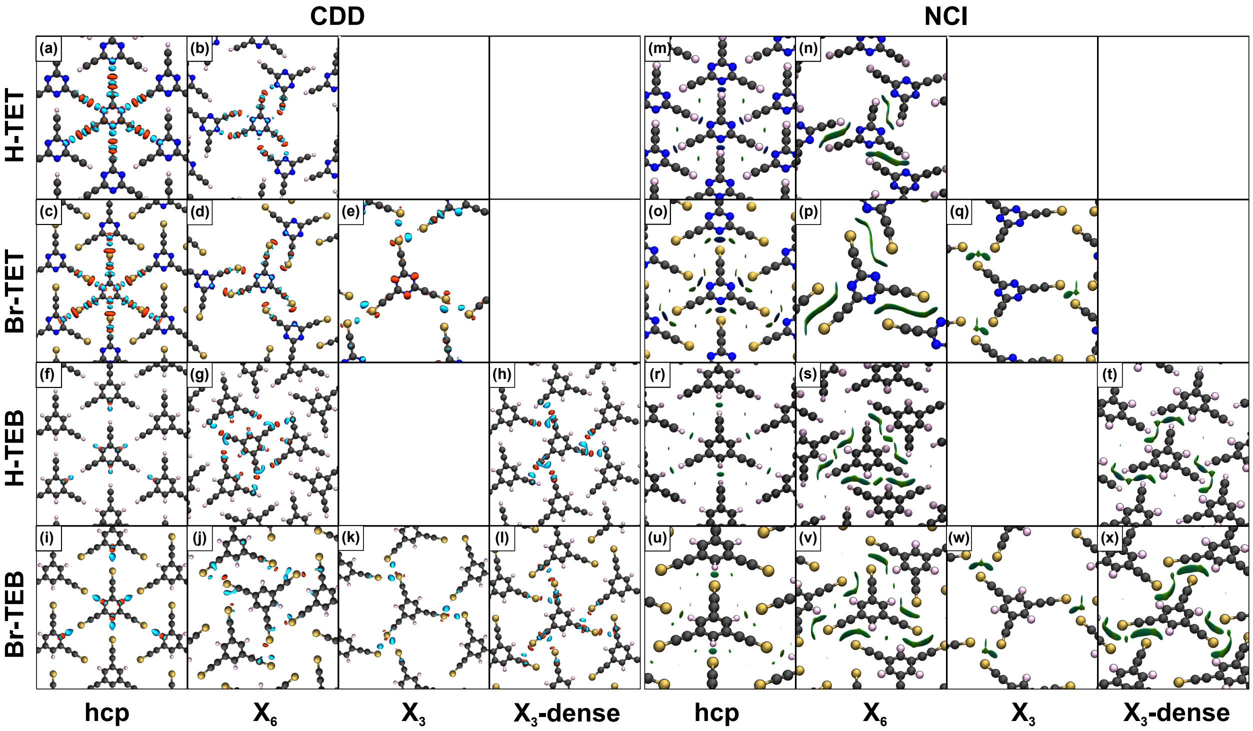

Figure S6: (a-l) CDD analysis and (m-x) NCI plots for H-TET, Br-TET, H-TEB, and Br-TEB comparing the hcp (first column), X_6-synthon (second column), X_3-synthon (third column) and dense X_3-synthon (fourth column) structures. Charge accumulation and depletion are displayed by cyan and orange iso-densities for values of $0.01\,\mathrm{e\AA^{-3}}$ for (a, b, c, d) and $0.005\,\mathrm{e\AA^{-3}}$ for (e, f, g, h, i, j, k, l). In NCI plots, attractive, repulsive, and weak noncovalent interactions are represented by a blue, red, and green color of the shown isosurface of $s = 0.5$, see the Computational Details in the main manuscript. NCI plots for the calculated geometries have an iso-value of 0.005. Color code: carbon, dark gray; nitrogen, blue; bromine, tan; hydrogen, rose.

Table SI1: Binding energies per molecule E_B in eV, primitive lattice constant $\mathbf{a}_0$, centre-to-centre distance d_{c-c}, distance $d_{t-\mathrm{N/H}}$ between the termination (t=H, Br) and the adjacent ring atom (N, H), and distance d_{t-t} between terminations for H-TET, Br-TET, H-TEB, and Br-TEB compared with experimental data given in brackets where possible. For hcp and X_3-synthon structures d_{c-c} equals $\mathbf{a}_0$ because of a single molecule per unit cell. All values are given in Å. For the H-TET, and H-TEB X_3-synthon structures are not stable and therefore neglected.

Structure	H-TET	Br-TET	H-TEB	Br-TEB
		hcp		
E_B	-0.69	-0.91	-0.08	-0.40
d_{c-c} / $\mathbf{a}_0$	8.5 (8.6-8.8)[1]	9.9 (10.0±0.5)	10.0	11.0
$d_{t-\mathrm{N/H}}$	2.1 (2.3±0.5)	2.8 (3.0±0.5)	2.4	2.7
d_{t-t}	4.5	5.2	5.0	5.5
		X_6-synthon		
E_B	-0.37	-0.42	-0.38	-0.45
$\mathbf{a}_0$	13.8	16.4	12.6	15.1 (15.0±0.5)
d_{c-c}	8.0	9.5	7.3	8.6 (8.9±0.5)
$d_{t-\mathrm{N/H}}$	2.5	3.2	2.7	3.0
d_{t-t}	4.1, 5.0	4.3, 6.1	3.1, 6.6	4.1, 6.9 (4.1±0.5)
		X_3-synthon		
E_B		-0.28		-0.33
d_{c-c} / $\mathbf{a}_0$		12.9		12.8
$d_{t-\mathrm{N/H}}$		6.6		5.6
d_{t-t}		3.7		3.5
		dense X_3-synthon		
E_B			-0.30	-0.33
d_{c-c} / $\mathbf{a}_0$			9.5	10.9
$d_{t-\mathrm{N/H}}$			2.7	3.1
d_{t-t}			2.8, 6.8	3.8, 7.1

[1] Range given by reference molecular crystal and the substrate supported case reported in this work.

References

(1) Yang, Z.; Gebhardt, J.; Schaub, T. A.; Sander, T.; Schönamsgruber, J.; Soni, H.; Görling, A.; Kivala, M.; Maier, S. *Nanoscale* **2018**, *10*, 3769–3776.
